# Sex-based differences in risk factors for incident myocardial infarction and stroke in the UK Biobank

**DOI:** 10.1093/ehjqcco/qcad029

**Published:** 2023-05-22

**Authors:** Elizabeth Remfry, Maddalena Ardissino, Celeste McCracken, Liliana Szabo, Stefan Neubauer, Nicholas C Harvey, Mamas A Mamas, John Robson, Steffen E Petersen, Zahra Raisi-Estabragh

**Affiliations:** William Harvey Research Institute, NIHR Barts Biomedical Research Centre, Queen Mary University of London, Charterhouse Square, London EC1M 6BQ, UK; National Heart and Lung Institute, Imperial College London, Hammersmith Hospital, London SW3 6LY, UK; Division of Cardiovascular Medicine, Radcliffe Department of Medicine, University of Oxford, National Institute for Health Research Oxford Biomedical Research Centre, Oxford University Hospitals NHS Foundation Trust, Oxford OX3 9DU, UK; William Harvey Research Institute, NIHR Barts Biomedical Research Centre, Queen Mary University of London, Charterhouse Square, London EC1M 6BQ, UK; Semmelweis University, Heart and Vascular Center, Hungary, Budapest 1122, Hungary; Barts Heart Centre, St Bartholomew's Hospital, Barts Health NHS Trust, West Smithfield EC1A 7BE, UK; Division of Cardiovascular Medicine, Radcliffe Department of Medicine, University of Oxford, National Institute for Health Research Oxford Biomedical Research Centre, Oxford University Hospitals NHS Foundation Trust, Oxford OX3 9DU, UK; MRC Lifecourse Epidemiology Centre, University of Southampton, Southampton SO16 6YD, UK; NIHR Southampton Biomedical Research Centre, University of Southampton and University Hospital Southampton NHS Foundation Trust, Southampton SO16 6YD, UK; Keele Cardiovascular Research Group, Keele University, Keele ST5 5BG, UK; Institute of Population Health, University of Manchester, Manchester M13 9NT, UK; Wolfson Institute of Population Health Sciences, Queen Mary University of London, London E1 4NS, UK; William Harvey Research Institute, NIHR Barts Biomedical Research Centre, Queen Mary University of London, Charterhouse Square, London EC1M 6BQ, UK; Barts Heart Centre, St Bartholomew's Hospital, Barts Health NHS Trust, West Smithfield EC1A 7BE, UK; Health Data Research UK, London NW1 2BE, UK; Alan Turing Institute, London NW1 2DB, UK; William Harvey Research Institute, NIHR Barts Biomedical Research Centre, Queen Mary University of London, Charterhouse Square, London EC1M 6BQ, UK; Barts Heart Centre, St Bartholomew's Hospital, Barts Health NHS Trust, West Smithfield EC1A 7BE, UK

**Keywords:** Risk factors, Sex differences, Myocardial infarction, Stroke

## Abstract

**Aim:**

This study examined sex-based differences in associations of vascular risk factors with incident cardiovascular events in the UK Biobank.

**Methods:**

Baseline participant demographic, clinical, laboratory, anthropometric, and imaging characteristics were collected. Multivariable Cox regression was used to estimate independent associations of vascular risk factors with incident myocardial infarction (MI) and ischaemic stroke for men and women. Women-to-men ratios of hazard ratios (RHRs), and related 95% confidence intervals, represent the relative effect-size magnitude by sex.

**Results:**

Among the 363 313 participants (53.5% women), 8470 experienced MI (29.9% women) and 7705 experienced stroke (40.1% women) over 12.66 [11.93, 13.38] years of prospective follow-up. Men had greater risk factor burden and higher arterial stiffness index at baseline. Women had greater age-related decline in aortic distensibility. Older age [RHR: 1.02 (1.01–1.03)], greater deprivation [RHR: 1.02 (1.00–1.03)], hypertension [RHR: 1.14 (1.02–1.27)], and current smoking [RHR: 1.45 (1.27–1.66)] were associated with a greater excess risk of MI in women than men. Low-density lipoprotein cholesterol was associated with excess MI risk in men [RHR: 0.90 (0.84–0.95)] and apolipoprotein A (ApoA) was less protective for MI in women [RHR: 1.65 (1.01–2.71)]. Older age was associated with excess risk of stroke [RHR: 1.01 (1.00–1.02)] and ApoA was less protective for stroke in women [RHR: 2.55 (1.58–4.14)].

**Conclusion:**

Older age, hypertension, and smoking appeared stronger drivers of cardiovascular disease in women, whereas lipid metrics appeared stronger risk determinants for men. These findings highlight the importance of sex-specific preventive strategies and suggest priority targets for intervention in men and women.

## Introduction

Cardiovascular disease (CVD) is a leading cause of morbidity and mortality worldwide, claiming 18.6 million lives each year.^1^ Important differences in the incidence, patterns, and outcomes of CVD have been described between men and women but the reasons for these differences are not completely understood.

Traditionally, CVDs were considered to predominantly affect men,^2^ which in part was due to a higher prevalence of some traditional risk factors (e.g. smoking) in men, but also to a historical under-representation of in women in health research.^3^ Understanding differences in sex-specific markers such as obstetric history, and variation in traditional risk factors is essential to optimizing CVD preventive strategies in men and women.^4^

Despite the substantial improvement made in narrowing the gap between sexes in cardiovascular outcomes over the past decades, existing evidence remains limited by restricted cohort sizes, lack of granularity in assessment of exposures such as lipid profiles, and for some risk factors such as smoking, contradictory results have been identified across populations.^5^ Moreover, the sex-based differences of more recently identified cardiovascular risk markers, such as ApoA, remain poorly understood.

The present study characterizes differences in distribution of cardiovascular risk factors between men and women in the UK Biobank cohort. These include traditional cardiovascular risk factors such as diabetes and hypertension, more novel risk factors such as ApoA, and emerging surrogate markers of cardiovascular risk such as aortic distensibility. Secondly, independent associations of a comprehensive host of risk factors with incident stroke and myocardial infarction (MI) are examined with a focus on defining sex-based differences in magnitudes of these associations.

## Methods

### Study population

The UK Biobank is a large population-based cohort that recruited over 500 000 people aged 40–69 years from across the UK between 2006 and 2010. Baseline assessment was performed according to a predefined protocol and included a touchscreen questionnaire, face-to-face interviews, a series of physical measures, and blood sampling.^6^ Incident health events are longitudinally tracked for all participants through electronic health record linkages with hospital admission and death registration data, with outcomes documented according to International Classification of Disease codes.^7,8^ Analysis was conducted on the set of participants for whom complete case data were available across the predefined set of exposures, outcomes, and covariates.

### Selection and ascertainment of covariates

Major risk factors were selected on basis of existing literature and biological knowledge of their role in risk of stroke and MI. The following factors were included: age, ethnicity, Townsend deprivation index,^9^ body mass index (BMI), systolic blood pressure (SBP), diastolic blood pressure (DBP), waist circumference, hip circumference, waist-hip ratio (WHR), glycated haemoglobin (HbA1c), total cholesterol, high-density lipoprotein cholesterol (HDL-C), low-density lipoprotein cholesterol (LDL-C), triglycerides, ApoA, apolipoprotein B (ApoB), smoking status, and diagnosed diabetes, hypertension and hypercholesterolaemia. All variables were extracted from baseline visit and were defined by UK Biobank field ID as identified in Supplementary material online, *Tables S1 and S2*.

### Arterial phenotyping

Arterial compliance has previously been reported to be an important marker for cardiovascular risk prediction.^10^ To obtain a unifying reflection of the dynamic trend in the cardiovascular risk profile across different ages and sexes, we evaluated two measures of arterial compliance: arterial stiffness index (ASI) and aortic distensibility (AoD). ASI provides an estimate of large artery stiffness and has been linked to incident cardiovascular events and mortality,^11^ whilst AoD provides an estimate of aortic compliance and is an indicator of local aortic bioelastic function and has been demonstrated to predict ischaemic events.^10^

Additional details regarding the extraction, outlier definition, and categorization of variables are provided in Supplementary Methods online.

### Ascertainment of outcomes

The primary outcomes were incident MI and incident stroke. Outcomes were ascertained using linked hospital and mortality data, as outlined in Supplementary material online, *Table S3*, over a mean of 12.66 [11.93, 13.38] years of prospective follow-up.

Individuals who had already experienced the outcome of interest prior to recruitment were excluded from analysis for that outcome. Individuals who suffered an endpoint during follow-up were left censored at the time of the event. In both analyses, individuals were right censored at date of death, or at the date of the last event reported in the UK Biobank (MI: 2021–11-12, stroke: 2021–10-25).

### Statistical analysis

This study is reported following the STROBE Statement guidelines.^12^ Baseline characteristics are presented for the whole cohort and stratified by sex as number (percentage) for categorical variables, mean (standard deviation) for normally distributed continuous variables, and median [interquartile range] for non-normally distributed continuous variables. Normality of distribution was ascertained by visual inspection of histograms.

The prevalence of prior MI and stroke at baseline visit, and subsequent incidence during follow-up were defined for the entire cohort and separately for each sex. Multivariable Cox proportional hazard regression models were used to obtain hazard ratios (HRs) and 95% confidence intervals (CIs) for each risk factor on the outcome. To calculate whether the HR differed between women and men, sex was added to the model as an interaction term to calculate RHRs, 95% CIs, and *P*-values.^13^ These are presented as women-to-men RHRs in all cases, a RHR > 1 demonstrates a greater proportional hazard increase in women, whilst a RHR < 1 indicates a greater proportional hazard increase in men.

Unadjusted models including only sex and age were conducted (Supplementary material online, *Table S4*). Multivariate models additionally included BMI, WHR, HbA1c, SBP, DBP, total cholesterol, HDL-C, LDL-C, triglycerides, ApoA, ApoB, and Townsend deprivation index as continuous variables. Abdominal obesity, smoking status, ethnicity, diabetes, high cholesterol, and hypertension were also included as categorical variables.

Arterial stiffness measures were not included as covariates. This is because these measures act as ‘proxy’ measures capturing the downstream vascular consequence of a range of adverse cardiometabolic factors which, in this study, is described by the host of cardiometabolic covariates that are already included in the model. The additional inclusion of arterial stiffness measures in the main models is therefore likely to be problematic from a causal perspective as it is likely to attenuate significant (and biologically causal) associations due to adjustment for a mediator.

Each model was assessed for multicollinearity to ensure variance inflation factors (VIF) for all covariates were less than 10. Each model was assessed for a violation of the proportional hazard assumption by visual assessment of scaled Schoenfeld residuals.

Poisson regression was used to obtain unadjusted incidence rates of stroke and MI per 1000 person years by sex. All analyses were performed using R version 4.2.1.^14^

## Results

### Baseline characteristics

**Figure 1:**
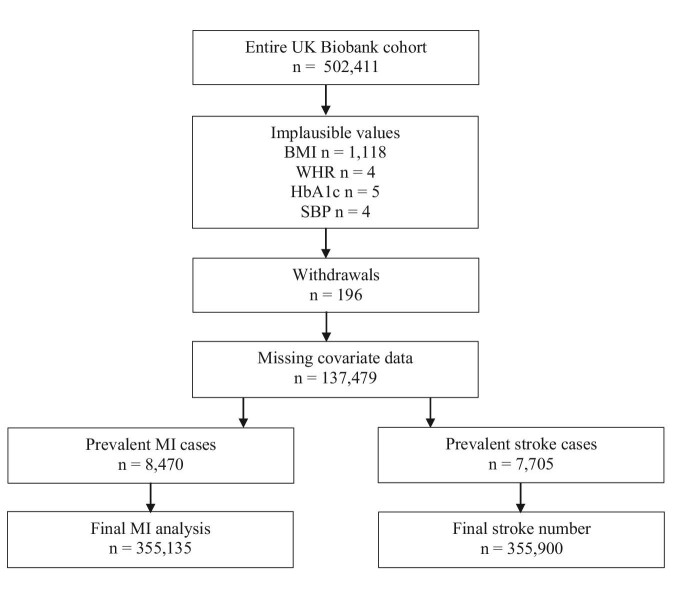
Flowchart of exclusion and inclusion. BMI: body mass index, WHR: waist-to-hip ratio, HbA1c: glycated haemoglobin, SBP: systolic blood pressure, MI: myocardial infarction.

The baseline characteristics of the 363 605 participants (53.8% female) included in the study are reported in *Table 1*. Missingness and reasons for exclusion are shown in *Figure 1*. At baseline visit, 8830 (19.6% female) participants reported a prior MI, and 6377 reported a previous stroke (40.9% female). In both men and women, the median age at enrolment was 58 years, 94.9% were of white ethnicity, and median Townsend deprivation index was –2.18 in women and –2.17 in men (i.e. more affluent that the UK national average).

**Table 1:** Characteristics of study cohort at baseline stratified by sex

	Overall	Female	Male
No (%)	363 605	195 542 (53.8)	168 063 (46.2)
Age (years)	58.00 [50.00, 63.00]	58.00 [50.00, 63.00]	58.00 [50.00, 64.00]
Ethnic group			
All other ethnic groups combined	18 574 (5.1)	9946 (5.1)	8628 (5.1)
White	345 031 (94.9)	185 596 (94.9)	159 435 (94.9)
Townsend deprivation index	–2.18 [–3.66, 0.43]	–2.18 [–3.65, 0.37]	–2.17 [–3.67, 0.50]
Smoking status			
Never	198 656 (54.6)	116 470 (59.6)	82 186 (48.9)
Previous	126 944 (34.9)	61 832 (31.6)	65 112 (38.7)
Current	38 005 (10.5)	17 240 (8.8)	20 765 (12.4)
Hypertension	107 814 (29.7)	50 301 (25.7)	57 513 (34.2)
Blood pressure (mmHg)			
Systolic blood pressure	136.00 [124.50, 149.50]	133.00 [121.00, 147.00]	139.00 [128.50, 151.50]
Diastolic blood pressure	82.23 (10.10)	80.65 (9.93)	84.07 (9.98)
Diabetes	19 455 (5.4)	7472 (3.8)	11 983 (7.1)
HbA1c (mmol/mol)	35.20 [32.80, 37.90]	35.20 [32.70, 37.70]	35.30 [32.80, 38.10]
BMI (kg/m^2^)	26.72 [24.13, 29.85]	26.08 [23.43, 29.64]	27.29 [24.98, 30.03]
Waist-to-hip ratio	0.87 (0.09)	0.82 (0.07)	0.94 (0.06)
Abdominal obesity			
Normal	182 633 (50.2)	134 536 (68.8)	48 097 (28.6)
Abdominal obesity	180 972 (49.8)	61 006 (31.2)	119 966 (71.4)
High cholesterol	72 296 (19.9)	29 209 (14.9)	43 087 (25.6)
Lipids			
Cholesterol (mmol/L)	5.70 (1.13)	5.88 (1.11)	5.49 (1.12)
HDL cholesterol (mmol/L)	1.40 [1.17, 1.68]	1.56 [1.33, 1.82]	1.24 [1.06, 1.45]
LDL direct (mmol/L)	3.56 (0.86)	3.63 (0.86)	3.49 (0.86)
Triglycerides (mmol/L)	1.48 [1.05, 2.14]	1.33 [0.96, 1.89]	1.69 [1.18, 2.44]
Apolipoprotein A (g/L)	1.51 [1.35, 1.70]	1.61 [1.45, 1.79]	1.41 [1.27, 1.56]
Apolipoprotein B (g/L)	1.02 [0.86, 1.18]	1.02 [0.87, 1.18]	1.01 [0.86, 1.18]
Aortic distensibility ascending (×10^−3^, mmHg^−1^) (% missing = 92.93)			
≤53 years	2.64 [1.88, 3.55]	2.66 [1.84, 3.66]	2.63 [1.92, 3.39]
54 to 69 years	1.27 [0.79, 1.99]	1.15 [0.69, 1.89]	1.42 [0.92, 2.08]
≥70 years	0.71 [0.48, 1.06]	0.60 [0.42, 0.88]	0.81 [0.56, 1.18]
Aortic distensibility descending (×10^−3^, mmHg^−1^) (% missing = 92.67)			
≤ 53 years	3.40 [2.68, 4.28]	3.42 [2.69, 4.31]	3.37 [2.66, 4.24]
54 to 69 years	2.30 [1.70, 3.04]	2.18 [1.58, 2.94]	2.42 [1.84, 3.14]
≥ 70 years	1.56 [1.16, 2.05]	1.40 [1.04, 1.83]	1.71 [1.29, 2.19]
Arterial stiffness index (m/s) (% missing = 65.03)	8.95 [6.86, 11.07]	8.20 [6.30, 10.37]	9.77 [7.72, 11.77]
Myocardial infarction			
Prevalent cases	8803	1728 (19.6)	7075 (80.4)
Incident cases	8470	2529 (29.9)	5941 (70.1)
Stroke			
Prevalent cases	6377	2610 (40.9)	3767 (59.1)
Incident cases	7705	3089 (40.1)	4616 (59.9)

g/L, grams per litre; HbA1c, glycated haemoglobin; HDL, high-density lipoprotein; kg/m^2^, kilograms metres squared; LDL, low-density lipoprotein; mmHg, millimetres of mercury; mmol/mol, millimoles per mole; mmol/L, millimoles per litre; m/s, metres per second; N, number. Results are mean (standard deviation), number (percentage) or median [interquartile range].

Men were more likely to be current or previous smokers compared to women (12.4% vs 8.8% current, and 38.7% vs 31.6% previous). Women had lower rates of diagnosed hypertension compared to men (25.7% vs 34.2%), as well as lower SBP (133 mmHg vs 139 mmHg) and DBP (81 mmHg vs 84 mmHg, respectively). The prevalence of diabetes was higher in men than women (7.1% vs 3.8%) although levels of HbA1c were similar across the sexes. Compared to men, women had a lower BMI (26.08 kg/m^2^ vs 27.29 kg/m^2^), smaller WHR (0.82 vs 0.94), and lower prevalence of abdominal obesity (31.2% vs 71.4%).

Men had higher rates of diagnosed hypercholesterolaemia than women (25.6% vs 14.9%). Compared to men, women had higher total cholesterol (5.88 vs 5.49 mmol/L), HDL-C (1.56 vs 1.24 mmol/L), LDL-C (3.63 vs 3.49 mmol/L), ApoA (1.61 vs 1.41 g/L), and ApoB (1.02 s 1.01 g/L) but lower triglycerides (1.33 vs 1.69 mmol/L).

Women had lower ASI than men at baseline (8.20 m/s vs 9.77 m/s). At younger ages (≤ 53 year), women and men had similar AoD at the ascending (2.66 vs 2.63 10^−3^ mmHg^−1^) and descending (3.42 vs 3.37 10^−3^ mmHg^−1^) aorta. However, at older ages (≥70 years), women had lower AoD at both the ascending (0.60 vs 0.81 10^−3^ mmHg^−1^) and descending (1.40 vs 1.71 10^−3^ mmHg^−1^) aorta than men.

### Myocardial infarction

**Figure 2:**
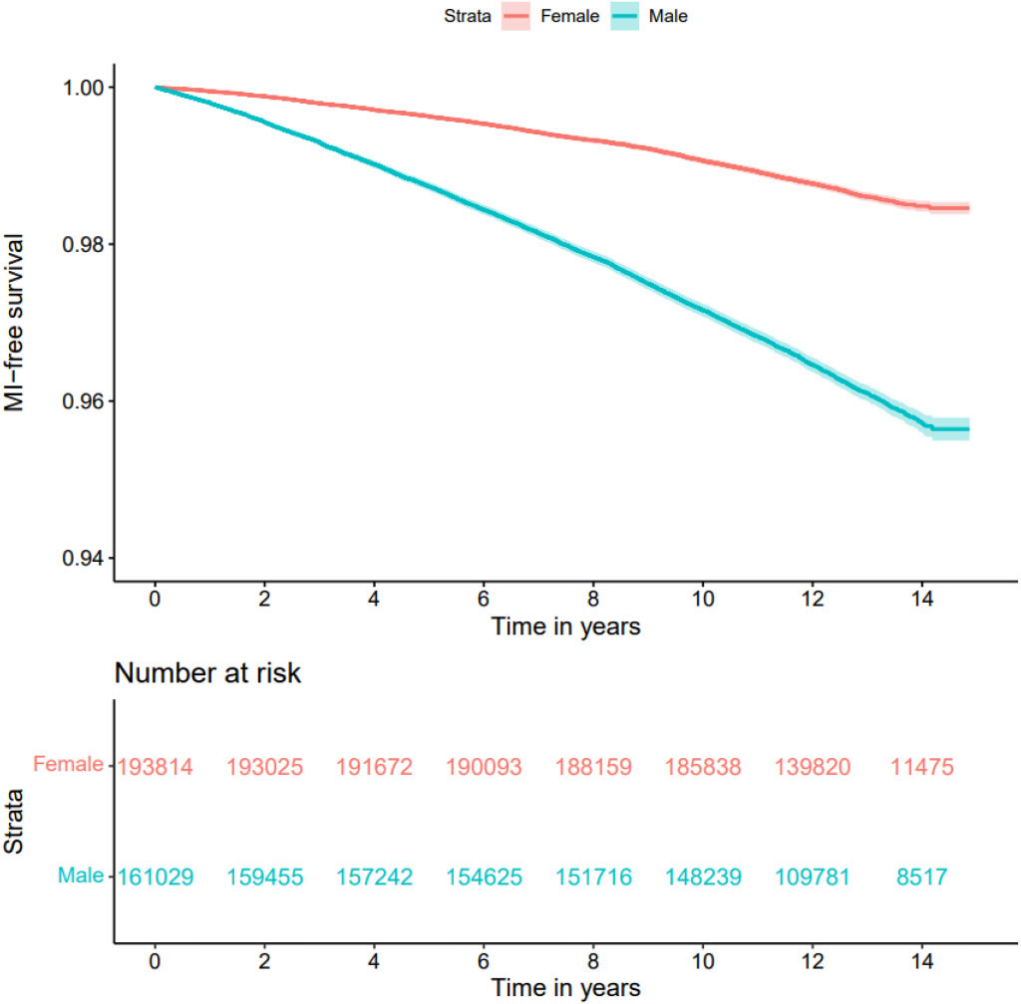
Unadjusted survival curve for myocardial infarction by sex.

During the study period, 8470 incident cases of MI were recorded, of which 29% occurred in women (*Figure 2*). The crude unadjusted incidence rate of MI per 1000 person years was 1.06 (95% CI 1.02–1.10) in women and 3.04 (95% CI 2.99–3.11) in men (Supplementary material online, *Table S5*). Men had a 2.8 times greater unadjusted hazard of incident MI than women (HR 2.81, 95% CI: 2.70–2.92, *P* < 0.001).

**Figure 3:**
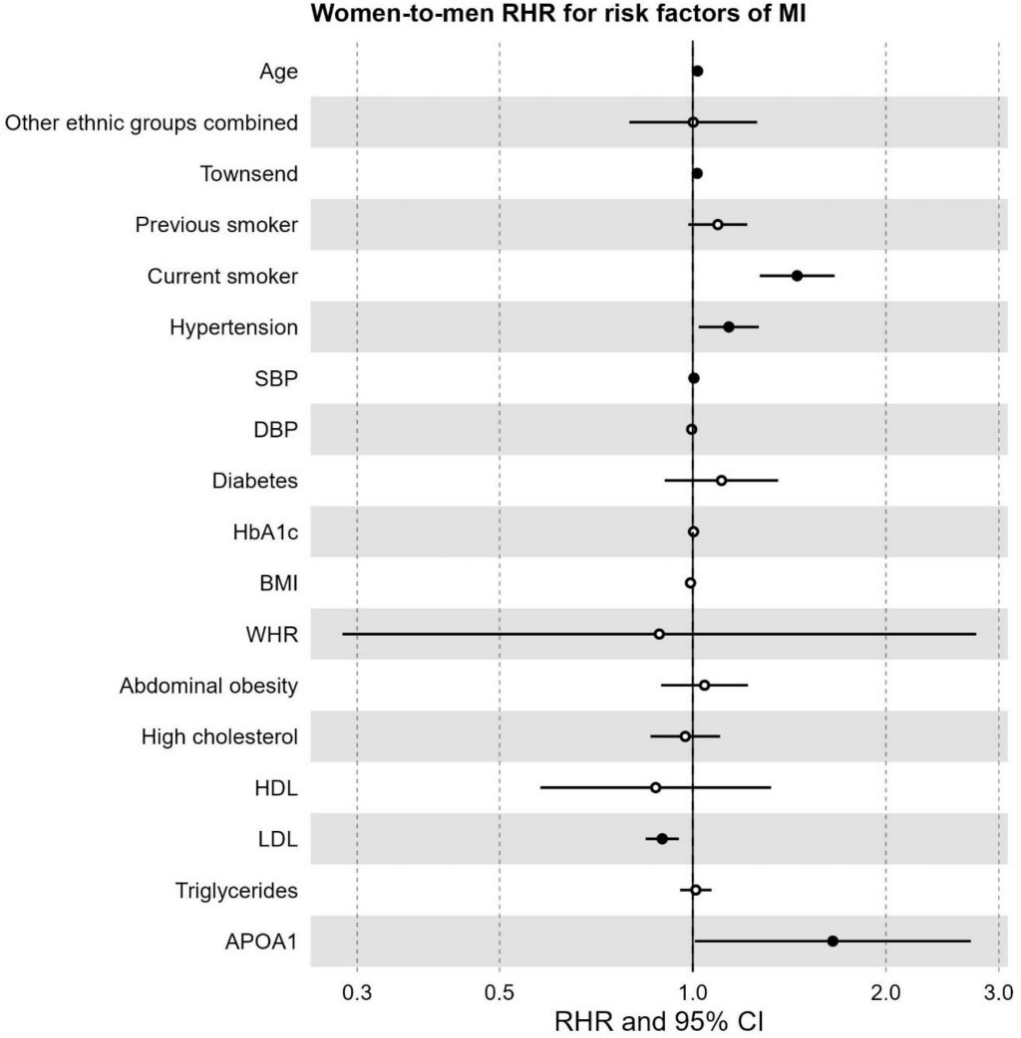
Forest plot of women-to-men ratio of hazard ratios for risk factors of MI.RHR: ratio of hazard ratios, CI: confidence interval, SBP: systolic blood pressure, DBP: diastolic blood pressure, HbA1c: glycated haemoglobin, BMI: body mass index, WHR: waist-to-hip ratio, HDL: high-density lipoprotein, LDL: low-density lipoprotein, APOA1: Apolipoprotein A. Significant risk factors displayed as solid points (*P*-value < 0.05).

Older age was associated with a higher hazard of MI in both sexes but conferred a greater hazard in women compared to men (RHR 1.02, 95% CI 1.01–1.03, *P* < 0.001). Similarly, greater deprivation was associated with a proportionally greater hazard of MI in women (RHR 1.02, 95% CI 1.00–1.03, *P* = 0.045), as was current smoking when compared to never smoking (RHR 1.45, 95% CI 1.27–1.66, *P* < 0.001). The presence of clinical hypertension was associated with a proportionally greater hazard in women (RHR 1.14, 95% CI 1.02–1.27, *P* = 0.019), and so was higher SBP (RHR 1.00, 95% CI 1.00–1.01, *P* = 0.014). In contrast, the association between higher LDL-C and MI was more pronounced in men, with a 10% relative increase in the hazard of MI (RHR 0.90, 95% CI 0.84–0.95, *P* < 0.001). Finally, the inverse association between ApoA and MI was stronger in men compared to women (RHR 1.65, 95% CI 1.01–2.71, *P* = 0.047). There was no indication of differential impacts of other risk factors on MI. The results are reported in *Table 2* and *Figure 3*.

**Table 2:** Hazard ratios for risk factors of MI by women and men, including women-to-men ratio of hazard ratio

	Women	Men	Women-to-men RHR
Covariates	HR (95% CI, *P* value)	HR (95% CI, *P* value)	RHR (95% CI, *P* value)
Age	1.06 (1.06–1.07, *P* < 0.001)	1.04 (1.04–1.05, *P* < 0.001)	1.02 (1.01–1.03, *P* < 0.001)
Ethnicity			
White	—	—	—
All other ethnic groups combined	1.01 (0.83–1.23, *P* = 0.924)	1.00 (0.89–1.13, *P* = 0.959)	1.00 (0.80–1.26, *P* = 0.988)
Townsend Deprivation Index	1.03 (1.02–1.04, *P* < 0.001)	1.01 (1.00–1.02, *P* = 0.005)	1.02 (1.00–1.03, *P* = 0.045)
Smoking status			
Never	—	—	—
Previous	1.22 (1.11–1.33, *P* < 0.001)	1.11 (1.05–1.18, *P* < 0.001)	1.09 (0.98–1.22, *P* = 0.096)
Current	2.72 (2.43–3.04, *P* < 0.001)	1.86 (1.73–2.00, *P* < 0.001)	1.45 (1.27–1.66, *P* < 0.001)
Hypertension	1.48 (1.35–1.62, *P* < 0.001)	1.30 (1.22–1.38, *P* < 0.001)	1.14 (1.02–1.27, *P* = 0.019)
Systolic blood pressure	1.02 (1.01–1.02, *P* < 0.001)	1.01 (1.01–1.01, *P* < 0.001)	1.00 (1.00–1.01, *P* = 0.014)
Diastolic blood pressure	0.99 (0.99–1.00*, *P* = 0.001)	1.00 (0.99–1.00, *P* = 0.008)	1.00 (0.99–1.00, *P* = 0.240)
Diabetes	1.45 (1.22–1.73, *P* < 0.001)	1.31 (1.18–1.45, *P* < 0.001)	1.11 (0.90–1.36, *P* = 0.321)
HbA1c	1.02 (1.01–1.02, *P* < 0.001)	1.01 (1.01–1.02, *P* < 0.001)	1.00 (1.00–1.01, *P* = 0.381)
BMI	0.99 (0.98–1.00, *P* = 0.030)	1.00 (0.99–1.01, *P* = 0.60	0.99 (0.98–1.00, *P* = 0.188)
Waist-to-hip ratio	2.55 (0.98–6.65, *P* = 0.055)	2.82 (1.53–5.22, *P* = 0.001)	0.89 (0.28–2.77, *P* = 0.836)
Abdominal obesity			
Normal	—	—	—
Abdominal obesity	1.04 (0.92–1.19, *P* = 0.508)	1.00 (0.92–1.09, *P* = 0.959)	1.04 (0.89–1.22, *P* = 0.595)
High cholesterol	1.31 (1.18–1.45, *P* < 0.001)	1.34 (1.26–1.44, *P* < 0.001)	0.97 (0.86–1.10, *P* = 0.673)
HDL cholesterol	0.64 (0.46–0.88, *P* = 0.006)	0.72 (0.56–0.93, *P* = 0.013)	0.88 (0.58–1.32, *P* = 0.529)
LDL cholesterol	1.30 (1.24–1.37, *P* < 0.001)	1.45 (1.40–1.50, *P* < 0.001)	0.90 (0.84–0.95, *P* < 0.001)
Triglycerides	1.01 (0.96–1.07, *P* = 0.566)	1.00 (0.98–1.03, *P* = 0.769)	1.01 (0.96–1.07, *P* = 0.704)
Apolipoprotein A	0.77 (0.52–1.14, *P* = 0.191)	0.47 (0.35–0.64, *P* < 0.001)	1.65 (1.01–2.71, *P* = 0.047)

BMI, body mass index; CI, confidence interval; HbA1c, glycated haemoglobin; HDL, high-density lipoprotein; HR, hazard ratio; LDL, low-density lipoprotein; RHR, ratio of hazard ratios.

### Stroke

**Figure 4:**
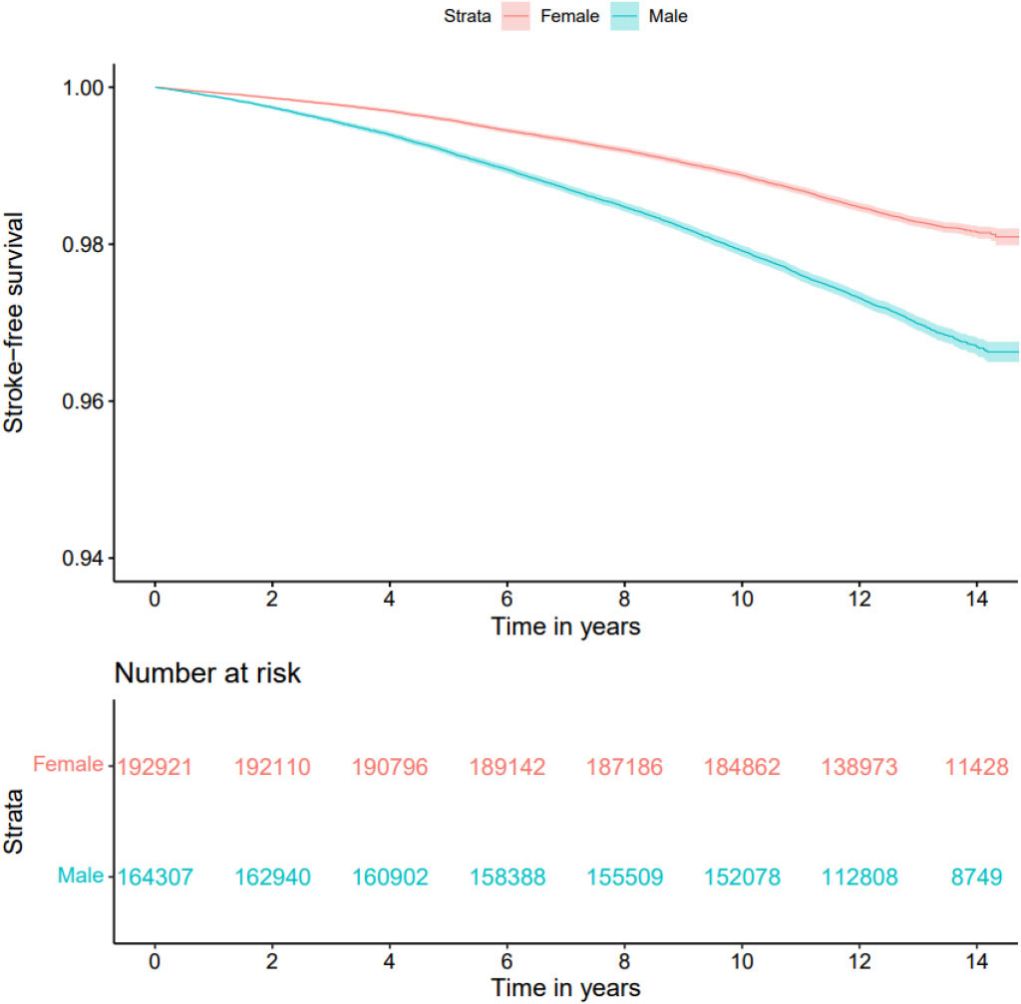
Unadjusted survival curve for stroke by sex.

A total of 7705 incident cases of stroke were recorded. Among these 40.1% occurred in women (*Figure 4*). The crude unadjusted incidence rate per 1000 person years was 1.30 (95% CI 1.26–1.35) for women, and 2.30 (95% CI 2.24–2.37) in men (Supplementary material online, *Table S5*). Overall, men had an unadjusted 1.7 times greater hazard of incident stroke than women (HR 1.73, 95% CI 1.67–1.80, *P* < 0.001).

**Figure 5:**
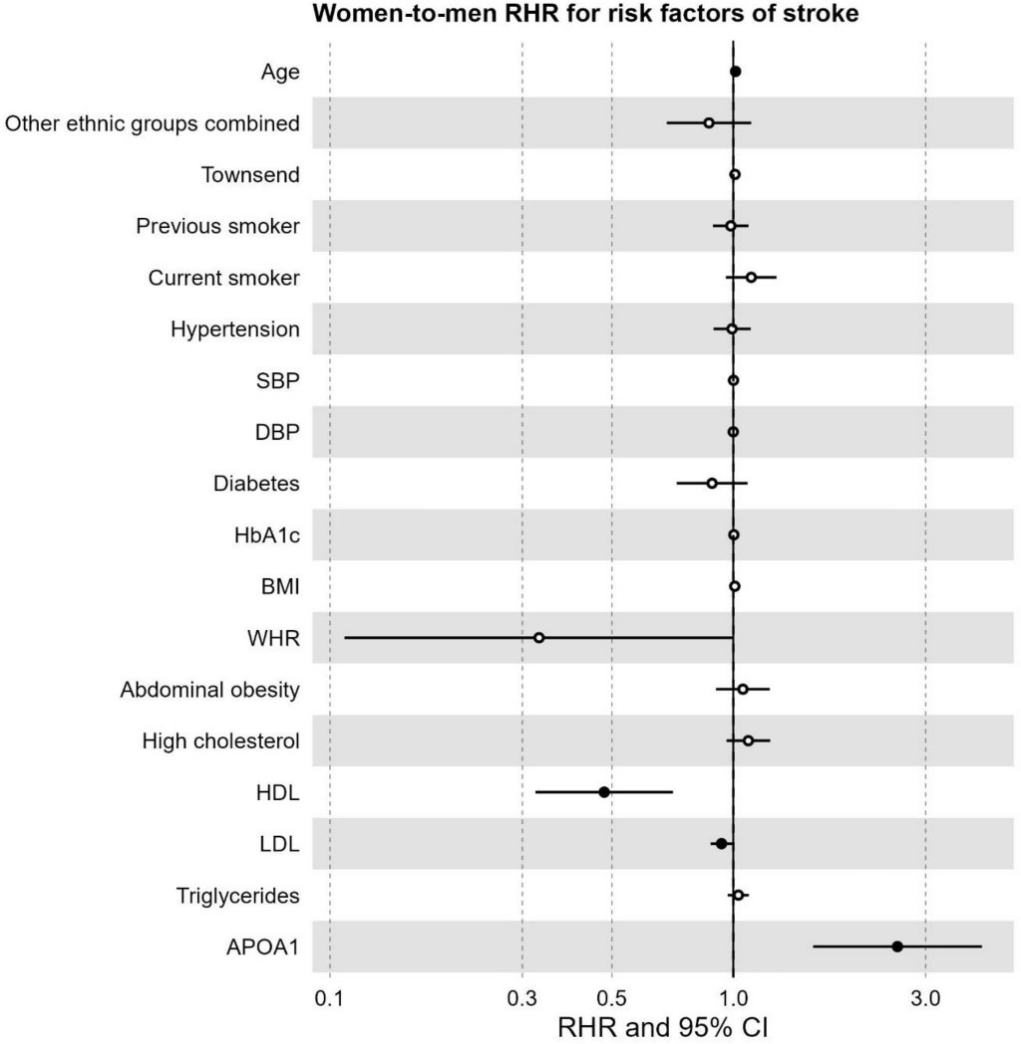
Forest plot of women-to-men ratio of hazard ratios for risk factors of stroke. RHR: ratio of hazard ratios, CI: confidence interval, SBP: systolic blood pressure, DBP: diastolic blood pressure, HbA1c: glycated haemoglobin, BMI: body mass index, WHR: waist-to-hip ratio, HDL: high-density lipoprotein, LDL: low-density lipoprotein, APOA1: Apolipoprotein A. Significant risk factors displayed as solid points (*P*-value < 0.05).

Older age was associated with proportionally greater hazard of stroke in women compared to men (RHR 1.01, 95% CI 1.00–1.02, *P* = 0.002). Conversely, higher HDL-C was more strongly associated with hazard of stroke in men, with a 52% proportionally greater hazard per unit HDL-C (RHR 0.48, 95% CI 0.32—0.71, *P* < 0.001). This was similar for LDL-C with a 6% proportionally greater hazard of stroke per unit LDL-C (RHR 0.94, 95% CI 0.88–1.00, *P* = 0.036). Finally, there was a large sex difference in ApoA (RHR 2.55, 95% CI 1.58–4.14, *P* < 0.001), suggesting a stronger protective effect in men compared to women. There was no indication of differential impacts of other risk factors on stroke. The results are reported in *Table 3* and *Figure 5*.

**Table 3:** Hazard ratios for risk factors of stroke by women and men, including women-to-men ratio of hazard ratio

	Women	Men	Women-to-men
Covariates	HR (95% CI, *P* value)	HR (95% CI, *P* value)	RHR (95% CI, *P* value)
Age	1.10 (1.10–1.11, *P* < 0.001)	1.09 (1.08–1.09, *P* < 0.001)	1.01 (1.00–1.02, *P* = 0.002)
Ethnicity			
White	—	—	—
All other ethnic groups combined	0.82 (0.68–1.00, *P* = 0.047)	0.95 (0.82–1.09, *P* = 0.463)	0.87 (0.68–1.11, *P* = 0.259)
Townsend Deprivation Index	1.04 (1.02–1.05, *P* < 0.001)	1.03 (1.02–1.04, *P* < 0.001)	1.01 (0.99–1.03, *P* = 0.199)
Smoking status			
Never	—	—	—
Previous	1.07 (0.99–1.16, *P* = 0.088)	1.09 (1.02–1.16, *P* = 0.013)	0.99 (0.89–1.09, *P* = 0.786)
Current	1.84 (1.64–2.07, *P* < 0.001)	1.66 (1.52–1.81, *P* < 0.001)	1.11 (0.96–1.28, *P* = 0.166)
Hypertension	1.37 (1.26–1.49, *P* < 0.001)	1.38 (1.29–1.48, *P* < 0.001)	0.99 (0.89–1.10, *P* = 0.896)
Systolic blood pressure	1.01 (1.01–1.01, *P* < 0.001)	1.01 (1.01–1.01, *P* < 0.001)	1.00 (1.00–1.00, *P* = 0.472)
Diastolic blood pressure	1.00 (1.00–1.01, *P* = 0.220)	1.00 (1.00–1.01, *P* = 0.126)	1.00 (0.99–1.01, *P* = 0.964)
Diabetes	1.16 (0.98–1.37, *P* = 0.088)	1.30 (1.16–1.46, *P* < 0.001)	0.89 (0.72–1.08, *P* = 0.241)
HbA1c	1.02 (1.01–1.02, *P* < 0.001)	1.02 (1.02–1.02, *P* < 0.001)	1.00 (1.00–1.01, *P* = 0.357)
BMI	1.00 (0.99–1.01, *P* = 0.650)	0.99 (0.98–1.00, *P* = 0.141)	1.01 (1.00–1.02, *P* = 0.165)
Waist-to-hip ratio	1.85 (0.77–4.43, *P* = 0.168)	5.62 (2.82–11.17, *P* < 0.001)	0.33 (0.11–1.00, *P* = 0.051)
Abdominal obesity			
Normal	—	—	—
Abdominal obesity	0.98 (0.87–1.10, *P* = 0.746)	0.93 (0.84–1.02, *P* = 0.134)	1.06 (0.91–1.23, *P* = 0.483)
High cholesterol	1.09 (0.99–1.20, *P* = 0.087)	1.00 (0.93–1.08, *P* = 0.991)	1.09 (0.96–1.23, *P* = 0.176)
HDL cholesterol	0.85 (0.64–1.13, *P* = 0.274)	1.78 (1.36–2.35, *P* < 0.001)	0.48 (0.32–0.71, *P* < 0.001)
LDL direct	0.96 (0.91–1.00, *P* = 0.065)	1.02 (0.98–1.07, *P* = 0.280)	0.94 (0.88–1.00, *P* = 0.036)
Triglycerides	1.00 (0.95–1.05, *P* = 0.970)	0.97 (0.94–1.00, *P* = 0.068)	1.03 (0.97–1.09, *P* = 0.346)
Apolipoprotein A	0.89 (0.63–1.26, *P* = 0.508)	0.35 (0.25–0.49, *P* < 0.001)	2.55 (1.58–4.14, *P* < 0.001)

BMI, body mass index; CI, confidence interval; HbA1c, glycated haemoglobin; HDL, high-density lipoprotein; HR, hazard ratio; LDL, low-density lipoprotein; RHR, ratio of hazard ratios.

### Missing data and multicollinearity assessment

ASI measurement was added to the UK Biobank protocol towards the end of recruitment (available for 35%). AoD is an image-derived metric and was available for the random subset of participants included in the UK Biobank Imaging Study (available for 7%). Given that these variables were not included in the main models, the impact of their missingness was not further assessed. For the variables included in the main model, a subanalysis comparing participants with complete data (72.37%) to those with missing data is reported in Supplementary material online, *Table S6*. The results of the analysis suggest that retained cases did not systematically differ from those with missing data.

VIF scores for variables scoring over 10 in the initial model are reported in Supplementary material online, *Table S7*. After removal of total cholesterol and ApoB from the model, all covariates had VIF score of less than 10.

## Discussion

This study demonstrates sex differences in major risk factors for MI and stroke. First, emerging risk markers, including LDL-C and ApoA, were more strongly associated with cardiovascular outcomes in men compared to women. Second, current smoking, socioeconomic deprivation, hypertension, and older age were associated with disproportionately greater increases in hazards of cardiovascular outcomes in women compared to men. Third, examination of arterial compliance measures, which have been previously found to predict cardiovascular events, validated a baseline higher risk in men but a steeper age-related trajectory of increasing risk in women.

### Lipid profiles

Previous studies have reported stronger associations between LDL-C and CVD in men. A prior Mendelian randomization study reported a 32% increased odds of CVD per 1 SD increase in genetically predicted LDL-C in women, with a corresponding 52% increased odds in men.^15^ Similarly, the observational PURE registry reported higher magnitudes of association of non-HDL-C traits with CVD in men.^5^

In this study, ApoA was associated with lower hazards of stroke and MI in men. Under causal assumptions, this suggests that ApoA might be less protective against CVD in women. This differential protective effect has been previously reported for MI,^16^ but not for stroke.^17^ This has important implications for the clinical investigation of interventions on ApoA aimed at reducing cardiovascular events and its use in risk prediction models.

Previous studies have highlighted inverse associations between HDL-C and CVD.^18–20^ This was replicated in this study for MI, but paradoxically for stroke we identified a direct association with a 52% relative higher hazard per unit increase in men. This result is likely due to the inclusion of ApoA in this model, which is a known component of the HDL-C particle. In a post-hoc analysis excluding ApoA, the previously reported inverse association between HDL-C and stroke in men was replicated (HR 0.83, 95% CI 0.74–0.93, *P* = 0.001). In line with previous studies which did not demonstrate benefit of HDL-C augmentation on risk of cardiovascular events,^21^ this result suggests that previously described ‘protective’ signals of HDL-C on cardiovascular events may be conveyed predominantly by ApoA.

### Age

Age was associated with an increased hazard of stroke and MI in women, compared with men. This is consistent with previous research that identified women experience their first stroke or MI event at older ages.^22,23^ This result was further validated by examining arterial compliance measures. Despite men having a higher baseline ASI, reflecting greater baseline cardiovascular risk, we observed a steeper age-related decline in AoD in women, which suggest a more rapid age-related increase in cardiovascular risk. Given the age demographic of this cohort, this increase in cardiovascular risk may occur after loss of the cardioprotective exposure to oestrogen. From a clinical perspective, this suggests that cardiovascular risk assessment and prevention strategies should be intensified with progressive age, particularly in women.

### Smoking status

This study identified a proportionally greater association between current smoking status and MI in women, in line with previous findings.^24,25^ The mechanism behind the excess risk in women is likely multifactorial. It might relate to differences in smoking patterns, or it might be conferred by higher rates of smoking continuation: women are less likely to receive counselling, to stop smoking, and on average quitters stop at an older age than men.^26–28^ Overall, the results highlight the key importance of smoking cessation in women and call for further research exploring whether the heterogeneity in hazards relates to biological or structural differences in healthcare systems.

### Hypertension

In this study, we identified a disproportionately higher hazard of MI associated with hypertension in women compared to men. The INTERHEART^22^ and PURE study^5^ have both previously reported similar findings. In this study, we did not identify any differences in hazards for stroke, though previous UK Biobank research found a higher risk in women only at higher stages of hypertension.^29^ There are multiple potential mechanisms behind this. Hypertension is known to be a major risk factor for hypertensive disorders of pregnancy^30^ and the development of the acute, severe cardiac, and endothelial dysfunction that ensues from these might act to heighten cardiovascular risk. Women with hypertension may also be treated differently to men, for example through avoidance of drug classes contraindicated in pregnancy.^31^ The results of this study provide evidence to support the growing view that sex-specific frameworks should be considered for screening, monitoring, and weighing of blood pressure as a component of global cardiovascular risk.^32^

### Socioeconomic status

The results of this study identified an association of larger magnitude between Townsend deprivation index and MI in women compared to men. Lower socioeconomic status (SES) is known to be associated with CVD,^33^ although in a previous study on the UK Biobank no sex difference was found.^34^ However, a large meta-analysis of more than 22 million participants found that low SES was associated with 34% excess risk of developing coronary heart disease in women compared to men.^35^ The mechanism behind this is unclear. As the Townsend deprivation index is an area-based rather than individual-based measure, the excess risk might reflect a greater relative deprivation among women compared to men within a single area. The findings might also reflect important inequities in access to healthcare with lower SES that might disproportionately impact women.

### Strengths and limitations

The key strength of this investigation lies in the inclusion of a broad set of risk factors including a detailed lipid profile of LDL-C, HDL-C, and ApoA, which highlighted the substantial differences in association across the sexes. Additionally, this study utilized a well-validated and intensely phenotyped population source, prospective outcome ascertainment with a substantial number of events ascertained through well-validated disease codes, and correlation of key findings with the novel cardiovascular risk marker of arterial compliance which further elucidate age-related cardiovascular risk trends.

Limitations of this study include the lack of diversity in ethnicity and SES in the UK Biobank, both of which may limit generalizability. The study population is also relatively healthy in comparison to the general public. The risk factor of smoking status was collected via self-report, which could lead to reporting bias. Finally, many of the lipid measures had more than 5% of data missing, though analysis of the characteristics of the individuals with missing data revealed no substantial systematic differences to the complete case cohort.

## Conclusions

The results of this study identify that smoking, low SES, and hypertension were more strongly associated with MI in women, whereas lipid traits were more strongly associated with both MI and stroke in men. Considering the historically male predominance in health research providing the basis for decisions made in everyday clinical practice, these results encourage further elucidation of sex-specific treatment effects in order to better inform clinical decision-making and treatment prioritization.

## Supplementary Material

qcad029_Supplemental_File

## Data availability statement

This research was conducted using the UK Biobank resource under access application 2964. UK Biobank will make the data available to all bona fide researchers for all types of health-related research that is in the public interest, without preferential or exclusive access for any persons. All researchers will be subject to the same application process and approval criteria as specified by UK Biobank. For more details on the access procedure, see the UK Biobank website: http://www.ukbiobank.ac.uk/register-apply.

## References

[1] Roth GA, Mensah GA, Johnson CO, Addolorato G, Ammirati E, Baddour LM et al. Global burden of cardiovascular diseases and risk factors, 1990–2019. J Am Coll Cardiol 2020;76:2982–3021. 33309175 10.1016/j.jacc.2020.11.010 PMC7755038

[2] Zhao M, Woodward M, Vaartjes I, Millett ERC, Klipstein-Grobusch K, Hyun K et al. Sex differences in cardiovascular medication prescription in primary care: a systematic review and meta-analysis. J Am Heart Assoc 2020;9:e014742. 32431190 10.1161/JAHA.119.014742 PMC7429003

[3] Barc J, Erdmann J. Sex matters? Sex matters! Cardiovasc Res 2022;118:e1–e3. 34927193 10.1093/cvr/cvab356

[4] Bairey Merz CN, Ramineni T, Leong D. Sex-specific risk factors for cardiovascular disease in women-making cardiovascular disease real. Curr Opin Cardiol 2018;33:500–505. 29965801 10.1097/HCO.0000000000000543

[5] Walli-Attaei M, Joseph P, Rosengren A, Chow CK, Rangarajan S, Lear SA et al. Variations between women and men in risk factors, treatments, cardiovascular disease incidence, and death in 27 high-income, middle-income, and low-income countries (PURE): a prospective cohort study. Lancet North Am Ed 2020;396:97–109. 10.1016/S0140-6736(20)30543-2 32445693

[6] UK Biobank . UK Biobank: protocol for a large-scale prospective epidemiological resource [cited 2022 Jun 7]. Available from: https://www.ukbiobank.ac.uk/media/gnkeyh2q/study-rationale.pdf.

[7] UK Biobank . Hospital inpatient data [Internet]. 2020 Aug [cited 2022 Jun 7]. Available from: https://biobank.ctsu.ox.ac.uk/crystal/crystal/docs/HospitalEpisodeStatistics.pdf

[8] UK Biobank . Mortality data: linkage to death registries [Internet]. 2020 Jun [cited 2022 Jun 7]. Available from: https://biobank.ndph.ox.ac.uk/showcase/showcase/docs/DeathLinkage.pdf

[9] Townsend P, Phillimore P, Beattie A. Health and Deprivation: Inequality and the North. London: Routledge; 1988. p. 240.

[10] Said MA, Eppinga RN, Lipsic E, Verweij N, van der Harst P. Relationship of arterial stiffness index and pulse pressure with cardiovascular disease and mortality. J Am Heart Assoc 2018;7:e007621. 29358193 10.1161/JAHA.117.007621 PMC5850166

[11] Pannier BM, Avolio AP, Hoeks A, Mancia G, Takazawa K. Methods and devices for measuring arterial compliance in humans. Am. J. Hypertens 2002;15:743–753. 12160200 10.1016/s0895-7061(02)02962-x

[12] von Elm E, Altman DG, Egger M, Pocock SJ, Gøtzsche PC, Vandenbroucke JP et al. The Strengthening the Reporting of Observational Studies in Epidemiology (STROBE) statement: guidelines for reporting observational studies. Ann Intern Med 2007;147:573–577. 17938396 10.7326/0003-4819-147-8-200710160-00010

[13] Woodward M. Rationale and tutorial for analysing and reporting sex differences in cardiovascular associations. Heart 2019;105:1701–1708. 31371439 10.1136/heartjnl-2019-315299 PMC6855792

[14] R Core Team . R: A Language and Environment for Statistical Computing. [Internet]. Vienna, Austria: R Foundation for Statistical Computing; 2022. Available from: https://www.R-project.org/.

[15] Cupido AJ, Asselbergs FW, Schmidt AF, Hovingh GK. Low-density lipoprotein cholesterol attributable cardiovascular disease risk is sex specific. J Am Heart Assoc 2022;11:e024248. 35699189 10.1161/JAHA.121.024248 PMC9238661

[16] Walldius G, Jungner I, Holme I, Aastveit AH, Kolar W, Steiner E. High apolipoprotein B, low apolipoprotein A-I, and improvement in the prediction of fatal myocardial infarction (AMORIS study): a prospective study. Lancet North Am Ed 2001;358:2026–2033. 10.1016/S0140-6736(01)07098-2 11755609

[17] O'Donnell MJ, McQueen M, Sniderman A, Pare G, Wang X, Hankey GJ et al. Association of Lipids, Lipoproteins, and Apolipoproteins with Stroke Subtypes in an International Case Control Study (INTERSTROKE). J Stroke 2022;24:224–235. 35677977 10.5853/jos.2021.02152 PMC9194539

[18] Sacco RL, Benson RT, Kargman DE, Boden-Albala B, Tuck C, Lin IF et al. High-density lipoprotein cholesterol and ischemic stroke in the elderly: The Northern Manhattan Stroke Study. JAMA 2001;285:2729–2735. 11386928 10.1001/jama.285.21.2729

[19] Wannamethee SG, Shaper AG, Ebrahim S. HDL-cholesterol, total cholesterol, and the risk of stroke in middle-aged British men. Stroke 2000;31:1882–1888. 10926951 10.1161/01.str.31.8.1882

[20] Sanossian N, Saver JL, Navab M, Ovbiagele B. High-density lipoprotein cholesterol. Stroke 2007;38:1104–1109. 17255541 10.1161/01.STR.0000258347.19449.0f

[21] Barter PJ, Caulfield M, Eriksson M, Grundy SM, Kastelein JJP, Komajda M et al. Effects of torcetrapib in patients at high risk for coronary events. N Engl J Med 2007;357:2109–2122. 17984165 10.1056/NEJMoa0706628

[22] Anand SS, Islam S, Rosengren A, Franzosi MG, Steyn K, Yusufali AH et al. Risk factors for myocardial infarction in women and men: insights from the INTERHEART study. Eur Heart J 2008;29:932–940. 18334475 10.1093/eurheartj/ehn018

[23] Reeves MJ, Bushnell CD, Howard G, Gargano JW, Duncan PW, Lynch G et al. Sex differences in stroke: epidemiology, clinical presentation, medical care, and outcomes. Lancet Neurol 2008;7:915–926. 18722812 10.1016/S1474-4422(08)70193-5 PMC2665267

[24] Lu Y, Li SX, Liu Y, Rodriguez F, Watson KE, Dreyer RP et al. Sex-specific risk factors associated with first acute myocardial infarction in young adults. JAMA Netw Open 2022;5:e229953. 35503221 10.1001/jamanetworkopen.2022.9953 PMC9066284

[25] Huxley RR, Woodward M. Cigarette smoking as a risk factor for coronary heart disease in women compared with men: a systematic review and meta-analysis of prospective cohort studies. Lancet North Am Ed 2011;378:1297–1305. 10.1016/S0140-6736(11)60781-2 21839503

[26] Peters SAE, Huxley RR, Woodward M. Do smoking habits differ between women and men in contemporary Western populations? Evidence from half a million people in the UK Biobank study. BMJ Open 2014;4:e005663. 10.1136/bmjopen-2014-005663 PMC4281541 25550291

[27] Dieleman LA, van Peet PG, Vos HMM. Gender differences within the barriers to smoking cessation and the preferences for interventions in primary care a qualitative study using focus groups in The Hague, The Netherlands. BMJ Open 2021;11:e042623. 10.1136/bmjopen-2020-042623 PMC7849885 33514579

[28] Rahmanian SD, Diaz PT, Wewers ME. Tobacco use and cessation among women: research and treatment-related issues. J Womens Health 2011;20:349–357. 10.1089/jwh.2010.2173 PMC3058892 21375414

[29] Dong C, Zhou C, Fu C, Hao W, Ozaki A, Shrestha N et al. Sex differences in the association between cardiovascular diseases and dementia subtypes: a prospective analysis of 464,616 UK Biobank participants. Biol Sex Differ 2022;13:21. 35526028 10.1186/s13293-022-00431-5 PMC9080133

[30] Ardissino M, Slob EAW, Millar O, Reddy RK, Lazzari L, Patel KHK et al. Maternal hypertension increases risk of preeclampsia and low fetal birthweight: genetic evidence from a Mendelian randomization study. Hypertension 2022;79:588–598. 35138876 10.1161/HYPERTENSIONAHA.121.18617 PMC7612410

[31] Kalibala J, Pechère-Bertschi A, Desmeules J. Gender differences in cardiovascular pharmacotherapy—the example of hypertension: a mini review. Front Pharmacol 2020;11:564. 32435193 10.3389/fphar.2020.00564 PMC7218117

[32] Gerdts E, Sudano I, Brouwers S, Borghi C, Bruno RM, Ceconi C et al. Sex differences in arterial hypertension. Eur Heart J 2022;43:4777–4788. 36136303 10.1093/eurheartj/ehac470 PMC9726450

[33] Hippisley-Cox J, Coupland C, Vinogradova Y, Robson J, Minhas R, Sheikh A et al. Predicting cardiovascular risk in England and Wales: prospective derivation and validation of QRISK2. BMJ 2008;336:1475–1482. 18573856 10.1136/bmj.39609.449676.25 PMC2440904

[34] Millett ERC, Peters SAE, Woodward M. Sex differences in risk factors for myocardial infarction: cohort study of UK Biobank participants. BMJ 2018;363:k4247. 30404896 10.1136/bmj.k4247 PMC6364292

[35] Backholer K, Peters SAE, Bots SH, Peeters A, Huxley RR, Woodward M. Sex differences in the relationship between socioeconomic status and cardiovascular disease: a systematic review and meta-analysis. J Epidemiol Community Health 2017;71:550–557. 27974445 10.1136/jech-2016-207890

