# Supplementary material for: Sex-based differences in risk factors for incident myocardial infarction and stroke in the UK Biobank
Source: Eur Heart J Qual Care Clin Outcomes. 2023 May 22;10(2):132–42. doi: 10.1093/ehjqcco/qcad029 (PMC10904726; doi:10.1093/ehjqcco/qcad029)
Supplement: qcad029_Supplemental_File [file qcad029_supplemental_file.docx]

# Supplementary Tables

## Supplementary Table 1 Ascertainment of covariates with associated UK Biobank code

| Covariate | UK Biobank Field ID |
| --- | --- |
| Socio-demographics |  |
| Age (years) | 21003 |
| Sex | 31 |
| Ethnicity | 21000 |
| Townsend deprivation index | 189 |
| Physical measurements |  |
| Systolic blood pressure (mmHg) | 4080 |
| Diastolic blood pressure (mmHg) | 4079 |
| Waist circumference (cm) | 48 |
| Hip circumference (cm) | 49 |
| Body mass index (kg/m^2^) | 21001 |
| Pulse wave arterial stiffness index | 21021 |
| Laboratory tests |  |
| HbA1c (mmol/mol) | 30750 |
| Total cholesterol (mmol/L) | 30690 |
| HDL cholesterol (mmol/L) | 30760 |
| LDL direct (mmol/L) | 30780 |
| Triglyceride (mmol/L) | 30870 |
| Apolipoprotein A (g/L) | 30630 |
| Apolipoprotein B (g/L) | 30640 |
| Risk factors |  |
| Smoking status | 20116 |
| Diabetes | As per Supplementary Table 2 |
| Hypertension | As per Supplementary Table 2 |
| High cholesterol | As per Supplementary Table 2 |

mmHg: millimetre of mercury, cm: centimetre, kg/m^2^: kilograms metres squared, HbA1c: glycated haemoglobin, mmol/mol: millimole per mole, mmol/L: millimole per litre, g/L: grams per litre

Supplementary Table 2 Ascertainment of clinical labels for hypertension, diabetes, and high cholesterol using ICD and UK Biobank codes

| Diabetes | UK Biobank Field ID | ICD Code | Description |
| --- | --- | --- | --- |
| Self-report | 20002 |  | Diabetes |
|  | 20002 |  | Type 1 diabetes |
|  | 20002 |  | Type 2 diabetes |
| Medication | 6177: 3 |  | Insulin |
|  | 6153: 3 |  | Medication for diabetes |
| Diagnosed by doctor | 2443 |  | Diabetes diagnosed by doctor |
|  | 2976 |  | Age diabetes diagnosed by doctor |
| Diagnoses - ICD9 | 41281  41271 | 250 | Diabetes mellitus |
| Diagnoses – ICD10 | 41280  41240 | E10 | Type 1 diabetes mellitus |
|  |  | E11 | Type 2 diabetes mellitus |
|  |  | E13 | Other specified diabetes mellitus |
|  |  | E14 | Unspecified diabetes mellitus |
|  |  | G590 | Diabetic mononeuropathy |
|  |  | G632 | Diabetic polyneuropathy |
|  |  | H280 | Diabetic cataract |
|  |  | H360 | Diabetic retinopathy |
|  |  | M142 | Diabetic arthropathy |
|  |  | N083 | Glomerular disorders in diabetes mellitus |
|  |  | O240 | Diabetes mellitus in pregnancy: Pre-existing type 1 diabetes mellitus |
|  |  | O241 | Diabetes mellitus in pregnancy: Pre-existing type 2 diabetes mellitus |
|  |  | O243 | Diabetes mellitus in pregnancy: Pre-existing diabetes mellitus, unspecified |
|  |  | O244 | Diabetes mellitus arising in pregnancy |
|  |  | O249 | Diabetes mellitus in pregnancy, unspecified |
|  |  | Y423 | Insulin and oral hypoglycaemic [antidiabetic] drugs |
| First occurrences | 130706 |  | Date E10 first reported (insulin-dependent diabetes mellitus) |
|  | 130708 |  | Date E11 first reported (non-insulin-dependent diabetes mellitus) |
|  | 130712 |  | Date E13 first reported (other specified diabetes mellitus) |
|  | 130714 |  | Date E14 first reported (unspecified diabetes mellitus) |
| High cholesterol |  |  |  |
| Self-report | 20002 |  | High cholesterol |
| Medications | 6177: 1 |  | Medication for cholesterol |
|  | 6153: 1 |  | Medication for cholesterol |
| Diagnoses - ICD9 | 41281  41271 | 272 | Disorders of lipoid metabolism |
| Diagnoses – ICD10 | 41280  41240 | E780 | Pure hypercholesterolaemia |
|  |  | E782 | Mixed hyperlipidaemia |
|  |  | E783 | Hyperchylomicronaemia |
|  |  | E784 | Other hyperlipidaemia |
|  |  | E785 | Hyperlipidaemia, unspecified |
| First occurrences | 130814 |  | Date E78 first reported (disorders of lipoprotein metabolism and other lipidaemias) |
| Hypertension |  |  |  |
| Self-report | 20002 |  | Essential hypertension |
|  | 20002 |  | Hypertension |
| Medication | 6177: 2 |  | Medication for blood pressure |
|  | 6153: 2 |  | Medication for blood pressure |
| First occurrences | 131286 |  | Date I10 first reported (essential (primary) hypertension) |
| Diagnosed by doctor | 2966 |  | Age high blood pressure diagnosed by doctor |
|  | 6150: 4 |  | High blood pressure diagnosed by doctor |
| Diagnoses - ICD9 | 41281  41271 | 4010 | Essential hypertension, specified as malignant |
|  |  | 4011 | Essential hypertension, specified as benign |
|  |  | 4019 | Essential hypertension, not specified as malignant or benign |
|  |  | 4039 | Hypertensive renal disease, not specified as malignant or benign |
| Diagnoses – ICD10 | 41280  41240 | I10 | Essential (primary) hypertension |
|  |  | I11.0 | Hypertensive heart disease with (congestive) heart failure |
|  |  | I11.9 | Hypertensive heart disease without (congestive) heart failure |
|  |  | I12.0 | Hypertensive renal disease with renal failure |
|  |  | I12.9 | Hypertensive renal disease without renal failure |
|  |  | I13.0 | Hypertensive heart and renal disease with (congestive) heart failure |
|  |  | I13.1 | Hypertensive heart and renal disease with renal failure |
|  |  | I13.2 | Hypertensive heart and renal disease with both (congestive) heart failure and renal failure |
|  |  | I13.9 | Hypertensive heart and renal disease, unspecified |
|  |  | I15.0 | Renovascular hypertension |
|  |  | I15.1 | Hypertension secondary to other renal disorders |
|  |  | I15.2 | Hypertension secondary to endocrine disorders |
|  |  | I15.8 | Other secondary hypertension |

ICD: International classification of diseases

Supplementary Table 3 Ascertainment of CVD outcomes, ICD and UK Biobank codes

| Source | UK Biobank Field ID | ICD Code | Description |
| --- | --- | --- | --- |
| Ischemic heart disease (myocardial infarction) | | | |
| Diagnoses - ICD9 | 41281  41271 | 410 | Acute myocardial infarction |
|  |  | 411 | Other acute and subacute forms of ischaemic heart disease |
|  |  | 412 | Old myocardial infarction |
| Diagnoses – ICD10 | 41280  41240 | I21 | Acute myocardial infarction |
|  |  | I22 | Subsequent myocardial infarction |
|  |  | I23 | Certain current complications following acute myocardial infarction |
| Self-report | 20002 |  | Heart attack/myocardial infarction |
| First occurrences | 131298 |  | Acute myocardial infarction |
|  | 131300 |  | Subsequent myocardial infarction |
|  | 131302 |  | Certain current complications following acute myocardial infarction |
| Diagnosed by doctor | 3894 |  | Age heart attack diagnosed |
|  | 6150: 1 |  | Heart attack diagnosed by doctor |
| Algorithm | 42000 |  | Date of myocardial infarction |
| Stroke | | | |
| Self-report | 20002 |  | Stroke |
|  |  |  | Ischaemic stroke |
|  |  |  | Brain haemorrhage |
| Diagnoses - ICD9 | 41281  41271 | 431 | Intracerebral haemorrhage |
|  |  | 4349 | Occlusion of cerebral arteries, unspecified |
| Diagnoses – ICD10 | 41280  41240 | I64 | Stroke, not specified as haemorrhage or infarction |
|  |  | I63 | Cerebral infarction |
|  |  | I61 | Intracerebral haemorrhage |
|  |  | I62 | Other nontraumatic intracranial haemorrhage |
| First occurrences | 131368 |  | Date I64 first reported (stroke, not specified as haemorrhage or infarction) |
|  | 131366 |  | Cerebral infarction |
|  | 131362 |  | Intracerebral haemorrhage |
|  | 131364 |  | other nontraumatic intracranial haemorrhage |
| Diagnosed by doctor | 4056 |  | Age stroke diagnosed |
|  | 6150: 3 |  | Stroke diagnosed by doctor |
| Algorithm | 42006 |  | Date of stroke |
|  | 42008 |  | Date of ischaemic stroke |
|  | 42010 |  | Date of intracerebral haemorrhage |

ICD: International classification of diseases

Supplementary Table 4 Unadjusted hazard ratios for MI and stroke, female as reference group

|  | Myocardial infarction | Stroke |
| --- | --- | --- |
| Covariates | HR (95% CI, p value) | HR (95% CI, p value) |
| Sex |  |  |
| Female | — | — |
| Male | 2.85 (2.72-2.99, p<0.001) | 1.73 (1.65-1.81, p<0.001) |
| Age | 1.06 (1.06-1.07, p<0.001) | 1.10 (1.10-1.11, p<0.001) |

Supplementary Table 5 Crude unadjusted incidence rates for MI and stroke

|  | Myocardial infarction | | Stroke | |
| --- | --- | --- | --- | --- |
|  | Women | Men | Women | Men |
| Number of events | 2,529 | 5,941 | 3,089 | 4,616 |
| Total person time | 2,386,905 | 1,956,541 | 2,375,017 | 2,003,980 |
| Incidence rate per 1000 person years | 1.06 | 3.04 | 1.30 | 2.30 |
| 95% CI | 1.02 – 1.10 | 2.99 – 3.11 | 1.26 - 1.35 | 2.24 - 2.37 |

CI: confidence intervals

Supplementary Table 6 Baseline characteristics for study cohort with complete cases and with missing data

|  | Participants missing data | Participants with complete cases |
| --- | --- | --- |
| N | 138,806 | 363,605 |
| Sex |  |  |
| Female | 77,783 (56.0) | 195,542 (53.8) |
| Male | 61,022 (44.0) | 168,063 (46.2) |
| Age (years) | 58.00 [50.00, 63.00] | 58.00 [50.00, 63.00] |
| BMI (kg/m^2^) | 26.80 [24.17, 30.03] | 26.72 [24.13, 29.85] |
| Waist-to-hip ratio | 0.87 (0.09) | 0.87 (0.09) |
| Ethnic group |  |  |
| All other ethnic groups combined | 8,447 (6.2) | 18,574 (5.1) |
| White | 127,580 (93.8) | 34,5031 (94.9) |
| Townsend deprivation index | -2.01 [-3.60, 0.89] | -2.18 [-3.66, 0.43] |
| Smoking status |  |  |
| Never | 74,818 (55.1) | 198,656 (54.6) |
| Previous | 46,080 (33.9) | 126,944 (34.9) |
| Current | 14,957 (11.0) | 38,005 (10.5) |
| HbA1c (mmol/mol) | 35.30 [32.80, 38.00] | 35.20 [32.80, 37.90] |
| Blood pressure (mmHg) |  |  |
| Systolic blood pressure | 136.00 [124.0, 149.0] | 136.00 [124.5, 149.5] |
| Diastolic blood pressure | 82.07 (10.22) | 82.23 (10.10) |
| Lipids |  |  |
| HDL cholesterol (mmol/L) | 1.38 [1.15, 1.66] | 1.40 [1.17, 1.68] |
| LDL direct (mmol/L) | 3.53 (0.90) | 3.56 (0.86) |
| Triglycerides (mmol/L) | 1.48 [1.04, 2.17] | 1.48 [1.05, 2.14] |
| Apolipoprotein B (g/L) | 1.02 [0.86, 1.18] | 1.02 [0.86, 1.18] |
| Diabetes | 8,378 (6.0) | 19,455 (5.4) |
| High cholesterol | 27,342 (19.7) | 72,296 (19.9) |
| Hypertension | 42,429 (30.6) | 10,7814 (29.7) |

N: number, kg/m^2^: kilograms metres squared, HbA1c: glycated haemoglobin, mmol/mol: millimoles per mole, mmol/L: millimoles per litre, HDL: high-density lipoprotein, LDL: low-density lipoprotein, g/L: grams per litre. Results are mean (standard deviation), number (percentage) or median [interquartile range]

## Supplementary Table 7 VIF score for variables scoring over 10

| Variable | VIF score |
| --- | --- |
| Cholesterol | 77.85 |
| Apolipoprotein B | 17.94 |
| LDL direct | 74.30 |

VIF: variance inflation factor, LDL: low-density lipoprotein

# Supplementary Methods

## Measurement and selection of covariates

Participant sex was obtained through linkage to NHS records and in a few cases was updated by the participant through self-report. Only the categories ‘Female’ and ‘Male’ were available. Age was taken when attending the assessment centre. Ethnic group was identified through the touchscreen self-report questionnaire, the available categories follow the 2001 census categories (1). These categories were combined to form the following groups: all other ethnic groups combined and white. Nomenclature for these groups were chosen based on UK government recommendations (2).

Townsend deprivation index was calculated based on the participant’s postcode. Participants were assigned a score based on the national census output areas, whereby lower values equate to less deprivation. Smoking status was identified through a self-report questionnaire, and categorised into 3 groups: current, previous and never.

Standing height was measured using a SECA 202 device (Seca GMBH, Germany), weight was taken using a Tanita BC-418 MA body composition analyser (3). Body mass index (BMI) was calculated by dividing the weight (kilograms) over the standing height (metres) squared. Participants with a BMI of over 50 or under 17 were excluded (n=1,118).

Waist and hip circumferences were measured using a SECA 200 measuring tape (Seca GMBH, Germany). Waist-to-hip ratio (WHR) was calculated by dividing the waist circumference (centimetres) by the hip circumference (centimetres). On visual inspection of WHR, extreme values were identified and cross referenced with BMI, extreme values (n=4) that did not match across the two variables were excluded. Abdominal obesity categories were selected using the WHO cut offs, a waist-to-hip ratio of 0.85 and larger for women and a waist-to-hip ratio of 0.90 and larger for men were considered as abdominally obese (4).

Glycated haemoglobin (HbA1c) was measured by HPLC analysis on a Bio-Rad VARIANT II Turbo. Values that sat outside of the manufacturers’ analytical range of 15-184 mmol/mol were excluded (n=5). Systolic blood pressure (SBP) and diastolic blood pressure (DBP) were measured automatically using the Omron HEM-7015IT digital blood pressure monitor. The measures were the average of two readings taken a few moments apart at baseline. Any results outside of the manufacturers range of 0 to 255 mmHg were excluded (n=4).

Cholesterol was measured by CHO-POD analysis on a Beckman Coulter AU5800, any values that sat outside of the manufacturer’s range of 0.5 to 18 mmol/L were excluded. HDL cholesterol was measured by enzyme immune-inhibition analysis on a Beckman Coulter AU5800. Any participants which were reported outside of the manufacturer’s analytical range of 0.05 - 4.65 mmol/L were excluded. LDL cholesterol was measured by enzymatic protective selection analysis on a Beckman Coulter AU5800. Any values reported outside of the manufacturer’s analytical range of 0.26 to 10.3 mmol/L were excluded.

Triglycerides were measured by GPO-POD analysis on a Beckman Coulter AU5800, any values outside of the manufacturer’s range 0.1 to 11.3 mmol/L were excluded. ApoA and ApoB were measured by immunoturbidimetric analysis on a Beckman Coulter AU5800. The manufacturer’s ranges were 0.4 to 2.5 g/L and 0.4 to 2 g/L respectively. Any values outside of these ranges were excluded.

Clinical diabetes, high cholesterol and hypertension status were recorded via both self-report methods and linkage to registry data as outlined in **Supplementary Table 3**. For diabetes during the touchscreen interview participants were asked whether they had had diabetes diagnosed by a doctor, age at diabetes diagnosis and the use of prescribed insulin. Using linked registry data any relevant ICD codes were extracted. First occurrence fields provided by the UK biobank were also utilised, these map to ICD10 codes from registry data.

High cholesterol was identified by self-reported disease or medication use, as well as relevant ICD codes and first occurrences from registry data. Hypertension was calculated through self-reported disease status, blood pressure medication, first occurrences and ICD codes.

Arterial stiffness index (ASI) was derived from finger plethysmography using a PulseTrace PCA2 device (CareFusion, USA), according to a pre- defined protocol (5). Outliers were removed from using the Tukey outlier removal protocol, as previously published using this dataset (6). ASI was added to the UK Biobank protocol towards the end of recruitment, resulting in data for 169,749 out of 500,000 participants. There was no criteria by which participants were selected for ASI measurement.

The UK Biobank Imaging Study, is aiming to provide cardiovascular magnetic resonance (CMR) imaging of a subset of 100,000 participants (7). Of which approximately 48,000 (July 2020) participants have been scanned. These scans are not clinically indicated and represent a random sample of the UK Biobank cohort. Aortic distensibility of the ascending aorta (10-3 mmHg-1) and the descending aorta (10-3 mmHg-1) was calculated as aortic strain per unit pressure extracted from cardiovascular magnetic resonance using a validated automated image analysis tool (8). Age at the imaging visit was used to categorise the cohort into three strata.

**References**

1. UK Government. List of ethnic groups [Internet]. [cited 2022 Nov 17]. Available from: https://www.ethnicity-facts-figures.service.gov.uk/style-guide/ethnic-groups

2. UK Government. Writing about ethnicity [Internet]. [cited 2022 Jul 3]. Available from: https://www.ethnicity-facts-figures.service.gov.uk/style-guide/writing-about-ethnicity

3. UK Biobank. UK Biobank: Protocol for a large-scale prospective epidemiological resource. p. 112.

4. World Health Organization. Waist Circumference and Waist–Hip Ratio: Report of a WHO Expert Consultation. Geneva, Switzerland; 2011 p. 39. Report No.: ISBN: 9789241501491.

5. UK Biobank. Arterial Pulse-Wave Velocity. 2011 p. 9.

6. Said MA, Eppinga RN, Lipsic E, Verweij N, van der Harst P. Relationship of Arterial Stiffness Index and Pulse Pressure With Cardiovascular Disease and Mortality. J Am Heart Assoc. 2018 Jan 22;7(2):e007621.

7. Petersen SE, Matthews PM, Bamberg F, Bluemke DA, Francis JM, Friedrich MG, et al. Imaging in population science: cardiovascular magnetic resonance in 100,000 participants of UK Biobank - rationale, challenges and approaches. J Cardiovasc Magn Reson. 2013 May 28;15(1):46.

8. Biasiolli L, Hann E, Lukaschuk E, Carapella V, Paiva JM, Aung N, et al. Automated localization and quality control of the aorta in cine CMR can significantly accelerate processing of the UK Biobank population data. PLOS ONE. 2019 Feb 14;14(2):e0212272.
